# Herzog is not required for mushroom body development or courtship learning & memory but is required for eye development in *Drosophila melanogaster*

**DOI:** 10.17912/micropub.biology.000720

**Published:** 2023-01-30

**Authors:** Madeleine J Palmer, Helen L Fitzsimons

**Affiliations:** 1 School of Natural Sciences, Massey University, Palmerston North, New Zealand

## Abstract

Herzog (Hzg, CG5830) shares similarity to members of the haloacid dehalogenase subfamily of small CTD phosphatases. In *Drosophila* it is a maternal gene essential for establishment of embryonic segment polarity, and oligomerization is required for activation of phosphatase activity. While Hzg is expressed in the brain, its role has not been investigated. To that end, we further characterized Hzg expression in the brain and found that it is highly expressed in neurons of the mushroom body where it localises to axons, and is also expressed in cortical glia. We investigated its role in mushroom body development as well as courtship learning and memory, but found that knockdown of Hzg had no impact on these processes. In contrast, knockdown in post-mitotic neurons in the eye resulted in disruption to ommatidial patterning and pigmentation, indicating it plays an important role in eye development.

**Figure 1. Expression of Hzg in the brain and phenotypic analysis following knockdown in the brain and eye.**
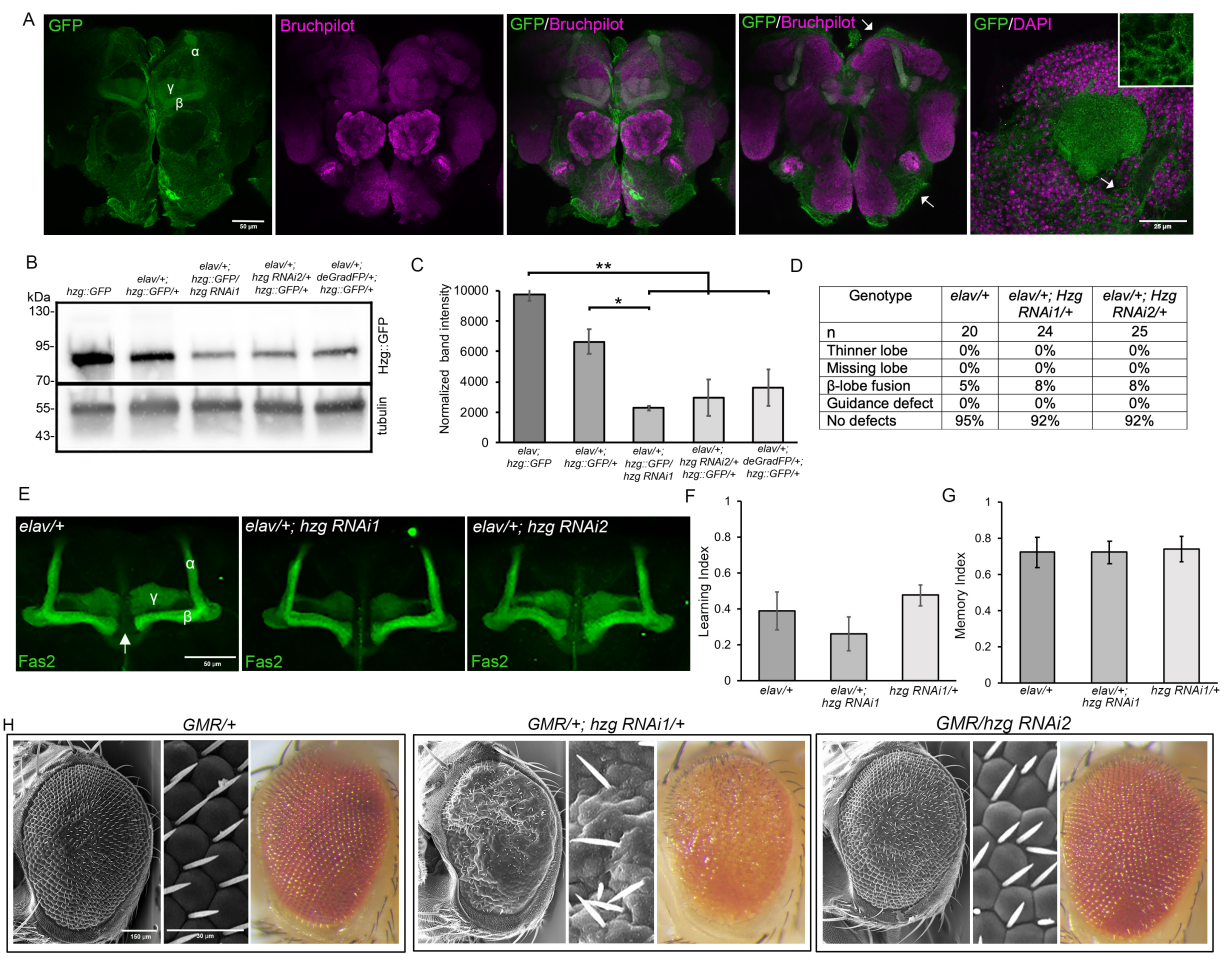
Fig 1A. Hzg::GFP brains were stained with anti-GFP and counterstained with either anti-Bruchpilot to label the synaptic neuropil, or with DAPI to label nuclei. The α, β and γ lobes of the mushroom body are indicated on the first image. Arrows point to Hzg::GFP in cortical glia. Inset: magnification of area under arrow showing Hzg::GFP in glia. B. Western blot of Hzg::GFP flies to evaluate efficacy of Hzg knockdown by expression of inverted repeat hairpins for RNAi ( *hzg RNAi1* and *hzg RNAi2* , or *deGradFP* . The GFP signal in lane 1 ( *hzg::GFP* homozygotes) is expected to be approximately twice that of lane two ( *hzg::GFP* heterozygotes). Lanes 2 to 5 are whole cell lysates from heads of progeny in which *elav-GAL4; hzg::GFP* females were crossed to either the *w(CS10)* control ( *elav/+; hzg::GFP/+* ), *UAS-RNAi* or *UAS-deGradFP* , as indicated. Hzg::GFP is estimated to be approximately 75 kDa. The blot was reprobed with α-tubulin as a loading control. The experiment was performed in triplicate and a representative blot is shown. C. Quantification of B. Mean Hzg::GFP band intensity (±SEM) normalized to α-tubulin is shown. (ANOVA F _(4,8)_ =17.91, p=0.00047, Tukey’s post hoc test *p<0.05, **p<0.01). D. *hzg RNAi1* or *hzg RNAi2* was expressed with *elav-GAL4* then immunohistochemistry with anti-Fas2 was performed on whole mount brains. The percentage of brains displaying each phenotype is shown. E. Brains displaying representative phenotypes are shown. The α, β and γ lobes of the mushroom body are indicated on the first image. These would have reduced thickness if the thinner lobe phenotype was present. The arrow indicates the midline between the hemispheres where the β lobes would cross and appear to join if the β lobe fusion phenotype was present. Guidance defects would be observed as the lobes projecting in a direction other than that seen in the control brain. F-G. Learning and immediate memory were assessed with the courtship suppression assay. *elav-GAL4* and *UAS-hzg RNAi1* flies were crossed to achieve pan-neuronal knockdown of *hzg* in progeny. Learning (F) was unaffected knockdown of *hzg* (ANOVA F _(2,52)_ =0.9, p=0.413), nor was (G) immediate memory (ANOVA F _(2,26)_ =0, p=1). H. Light and scanning electron micrographs of *Drosophila* eyes in which *hzg* was knocked down. The genotypes indicated in each panel were generated by crossing *GMR-GAL4* females to males carrying *UAS-hzg RNAi1, UAS-hzg RNAi2* or to the *w(CS10)* control ( *GMR/+* ). Representative images of n=20 eyes/group are shown.

## Description

Herzog (Hzg, CG5830) shares similarity to members of the haloacid dehalogenase subfamily of small CTD phosphatases, and has the highest homology with CTDSP1, which is a nuclear protein expressed in non-neuronal cells where it is required for silencing of neuronal genes (Yeo et al. 2005). Hzg remained uncharacterised in *Drosophila* until a recent study revealed that it is a maternal gene essential for embryogenesis. In embryos, Hzg is enzymatically inactive in its monomeric form until gastrulation at which time it oligomerizes via a prion-like domain into amyloid-like aggregations. This promotes association with the plasma membrane and activation of the phosphatase domain and is required for establishment of embryonic segment polarity (Nil et al. 2019). Despite these new insights into its role in embryogenesis and divergence in function from CTDSP1, whether Hzg function is required during other developmental stages or within specific adult tissues remains unknown. Curiously, analysis of Hzg expression via an Hzg::GFP protein trap, which contains an in-frame insertion of GFP into the endogenous *hzg* gene (Nagarkar-Jaiswal et al. 2015), revealed that it is expressed in neurons in the adult brain (Fig 1A). Hzg was detected widely throughout the brain, with particularly high expression in the mushroom body lobes, which are bundled axons of Kenyon cells, the intrinsic neurons of the mushroom body. In other areas of the brain, Hzg appeared in a mesh-like pattern (Fig 1A arrows) that is typical of cortical glia that enwrap neuronal cell bodies (Awasaki et al. 2008).

Given the strong expression in the mushroom body, we sought to examine whether Hzg was required for its development. Hzg was pan-neuronally knocked down in the brain via RNA interference with two independent inverted repeat hairpins. We also depleted Hzg via the deGradFP system, in which NSlmb-vhhGFP4 (a fusion between the vhh-GFP4 nano-body and the N-terminus of the F-box protein Slmb) binds GFP-tagged proteins and recruits the poly-ubiquitination machinery for proteasomal degradation (Caussinus and Affolter 2016). Flies carrying the pan-neuronal *elav-GAL4* driver in the *hzg::GFP* genetic background were crossed to flies carrying *UAS-hzg RNAi1* or *UAS-hzg RNAi2* as well as to *UAS-deGradFP* , and the level of Hzg::GFP in the heads of progeny was quantified via western blotting (Fig 1B,C). While they all significantly reduced Hzg, the two RNAi lines facilitated more efficient knockdown than deGradFP, therefore they were used to determine whether depletion of Hzg impacts mushroom body development. The mushroom body consists of approximately 2200 Kenyon cells, of which there are three subtypes; the α/β, α'/β' and γ neurons. The cell bodies of Kenyon cells are clustered at the posterior of the brain. Their axons are bundled together into a fibre termed the peduncle which projects towards the anterior of the brain where the axons split into the lobes. The γ neurons form the γ lobe which projects laterally, the α/β and α'/β' axons bifurcate vertically to form the α and α' lobes and horizontally to form the β and β' lobes, respectively (Crittenden et al. 1998). Defects in axon morphogenesis include missing lobes, thin lobes or fused lobes, indicating defects in axon elongation and termination, or guidance defects observed as mistargeted axons (Freymuth and Fitzsimons 2017). These defects are easily visualised and scored via immunohistochemistry for Fasciclin 2, which highlights the α β and γ lobes (Crittenden et al. 1998) and thus are ideal for assessment of defects in brain development.

None of these defects were observed following pan-neuronal knockdown of Hzg aside from two instances of minor β lobe fusion that were also observed in the control (Fig 1D,E). These data indicates that wildtype levels of Hzg are not required for mushroom body development, however the fact that it is robustly expressed in the adult brain suggested it may play a post-developmental role. In addition, RNA sequencing to identify transcriptional changes in the mushroom body after memory formation showed a four-fold increase in *hzg* expression after one hour of courtship training and learning, which also suggests a possible role in memory formation (Jones et al. 2018). To that end we investigated whether pan-neuronal depletion of Hzg impacted learning or memory via the courtship suppression assay. This assay tests whether a male fly learns and remembers rejection behavior from a female and in response, reduces his courtship efforts towards future females (Ejima and Griffith 2011, Raun et al. 2021). Following knockdown of Hzg via expression of *hzg RNAi1* in all neurons with *elav-GAL4* , no significant effect on learning (Fig 1F) or short-term memory (Fig 1G) was observed.

The *Drosophila* compound eye is made up of 800 individual ommatidia, each of which contain eight photoreceptor neurons, four cone cells and two primary pigment cells (Ready et al. 1976). Due to the precision required for development of the highly organised ommatidial arrays, the eye is particularly vulnerable to perturbation of molecular pathways required for development. Developmental abnormalities are visualised as ommatidial disorganisation and fusion, malformation of interommatidial bristles, loss of pigmentation and/or necrosis (Iyer et al. 2016). In addition, as photoreceptors employ many of the same molecular pathways as CNS neurons, they are an ideal model for the study of the biological processes that underpin neuronal development (Thomas and Wasserman 1999). To that end, we investigated whether Hzg is required for morphogenesis or survival of photoreceptors. Each of the *hzg* RNAi constructs were expressed with the *GMR-GAL4* driver, which promotes expression in all post-mitotic cells posterior to the morphogenetic furrow in the eye (Freeman 1996), and eyes were examined via light and scanning electron microscopy. Depletion of Hzg resulted in a rough eye phenotype with severe ommatidial fusion, disruption of bristles and reduced pigmentation (Fig 1H). In addition, all eyes expressing *hzg RNAi1* showed buckling of the fused surface of the eye. While h *zg RNAi2* also induced defects in ommatidia and bristle organisation, the defects were less severe, which is consistent with the less efficient knockdown.

Together these data show that depletion of Hzg in all neurons of the brain does not disrupt normal mushroom body development, nor impact courtship behavior, learning or memory. In contrast, reduced expression in the developing eye severely disrupted normal development, and further analysis of how Hzg regulates eye development is warranted, as is investigation of the role of Hzg in glia.

## Methods

**Fly strains**

All flies were raised at 25°C on standard medium on a 12 hour light/dark cycle unless otherwise indicated. Fly strains were obtained from the Bloomington Drosophila Stock Center or the Vienna Drosophila Resource Center as indicated in the reagents table.

**Immunohistochemical staining**

Whole flies were first pre-fixed in PFAT-DMSO (4% paraformaldehyde in 1X PBS +0.1% Triton X-100 + 5% DMSO) for one hour. Brains were dissected in 1X PBST (1X PBS + 0.5% Triton X-100) then fixed for 20 minutes in PFAT-DMSO. They were then transferred to 100% methanol and stored at -20°C. The brains were rehydrated for 5 minutes in 1:1 methanol, washed for 3 x 5 minutes in PBST, then were blocked for two hours in 5% normal goat serum in 1xPBST (1xPBS + 0.1% Triton X-100). Brains were incubated overnight at room temperature in primary antibody diluted in 5% normal goat serum in 1xPBST. Following 3 x 5 minute washes in PBST, brains were incubated overnight at 4°C with the appropriate secondary antibody in 5% normal goat serum in 1xPBST. For DAPI staining, brains were washed for 3 x 5 minutes in PBST after secondary antibody and incubated with DAPI (1:20,000 dilution in PBST) for 10 minutes before washing 3 x 20 minutes in PBST. The brains were mounted on a slide in Anti-fade (90% Glycerol, 0.2% n-propyl gallate in 1xPBS and kept at 4°C in the dark until they were ready to be imaged.

**Western blotting**

Flies were snap frozen and heads were removed by vortexing then separated from the bodies with a paintbrush on a piece of transparency over dry ice. Heads were homogenized in RIPA buffer (150 mM sodium chloride, 0.1% Triton X-100, 0.5% sodium deoxycholate, 0.1% SDS, 50 mM Tris, pH 8.0), then centrifuged for two minutes at 13,000g at 4°C and the supernatant was collected. The protein concentration of lysates was then quantified using the Pierce BCA Assay Kit (Thermo Fisher Scientific). For SDS-PAGE, 30 μg of lysate was suspended in 1x Laemmli buffer (2% sodium dodecyl sulphate, 5% 2-mecaptoethanol, 10% glycerol, 0.01% bromophenol blue, 60 mM Tris HCl pH 6.8). Samples were boiled for five minutes at 95ºC, then loaded onto a 4-20% SDS-PAGE gel and resolved at 180 V for 40 minutes in 1x Running buffer (25 mM Tris, 190 mM glycine, 0.1% SDS). The gel was then transferred to nitrocellulose membrane for 1 hour at 100V in chilled 1x Transfer buffer (25 mM Tris, 190 mM glycine, 0.1% SDS, 20% methanol). The membrane was blocked for 3 hours at room temperature in blocking buffer (5% skim milk powder in TBST [1x TBS, 0.1% Tween-20]) then washed 3 x 5 minutes in TBST. The membrane was then incubated in primary antibody in 1% skim milk powder in TBST, overnight at 4°C. The membrane was washed 3 x 5 mins with 1x TBST and then incubated for one hour at room temperature with secondary horseradish-peroxidase conjugated antibody in 1% skim milk powder in TBST. The membrane was washed three times for five minutes each using TBST. Detection was then performed using the ECL Prime (GE) reagent and imaged using the Azure c600 Gel Imaging System (Azure Biosystems).

**Confocal microscopy imaging**

Brains were imaged on a Leica TCS SP5 Confocal Laser Scanning Microscope. Optical sections (1 μm for whole brains and 0.5 μm through the mushroom body) were taken and z-stack images were collected through the appropriate brain regions. Images were processed using the ImageJ to generate maximal intensity projections.

**Scanning electron microscopy**

Flies were placed in a vial containing a disk of paper soaked with water overnight allowing them to clean their eyes of any food residue. Flies were then fixed in a modified Karnovsky’s fixative (3% glutaraldehyde 2% formaldehyde in 0.1 M phosphate buffer, pH 7.2, with Triton X-100) for minimum of 8 hours, and then washed and dehydrated in graded ethanol before being dried using liquid CO _2_ in Polaron E3000 series II critical point drying apparatus.

After fixation and drying, the heads of each fly were removed and collected. The heads were mounted on aluminium stubs with one eye facing outwards. Each stub was then coated in gold using the Baltec SCD 050 sputter coater for 200 seconds. Once the stubs were coated they were then stored in a moisture regulated sample safe until imaging was carried out. Imaging was carried out on the FEI Quanta 200 Environmental Scanning Electron Microscope at an accelerating voltage of 20 kV.

**Courtship suppression assay**

The repeat training courtship suppression assay was used to assess learning and memory. A male fly learns rejection behaviour when exposed to a mated female, and when tested with a new female, it reduces its courtship behavior, which can be quantified to provide a measurement of a fly’s capacity to learn and remember. The detailed methodology has been described previously (Freymuth and Fitzsimons 2017, Main et al. 2021). Virgin males of the test genotypes were collected, and each was housed individually so that their first exposure to a mature fly was the freshly mated female. To generate freshly mated females, 25 virgin females and 30 virgin males were mated for 48 hours. The morning of testing, the females and males were separated via brief CO _2_ anesthesia. Males were discarded and females were placed into a new vial and given a least an hour to recover. To assess learning, virgin males of the test genotype were placed into a testing chamber with a freshly mated female under a GoPro Hero 6 camera. Courtship behaviours recorded were orientating, ‘singing’, tapping, licking, attempted copulation and chasing, and the the proportion of time each male fly spent courting during the first and last 10 minutes of the hour was determined. A learning index (LI) was calculated for each male by the following equation: LI = 1-(CI of last 10 minutes/CI of first 10 minutes). A learning index of zero suggests no learning occurred, with higher numbers indicating increased learning.

For assessment of immediate memory, each male was placed in a training chamber with a freshly mated female for an hour during which time the male attempted to court the unreceptive female. An equivalent number of sham males were individually housed in training chambers and remained alone until testing. To test the memory of each trained or sham male, a freshly mated female was placed into a testing chamber with each male and courtship activity was recorded for 10 minutes. The proportion of time spent courting (courtship index, CI) of the test vs sham males was compared and used to calculate a memory index by the following equation: MI = 1-(CI of each trained fly/mean of sham group). The memory index gives a score between zero and one, with zero indicating no memory and higher numbers indicating increased memory. In each experiment, 16 to 20 flies were tested per genotype. In all experiments, the scorer was blind to the genotype of the flies. The test genotypes ( *elav-GAL4/+;UAS-hzg* *RNAi/+* ) were always tested alongside matched parental controls for the driver ( *elav-GAL4/+* ) and the RNAi line ( *UAS-hzg* *RNAi/+* ).

**Statistical analysis**

Statistical significance was assessed via one-way ANOVA followed by a post-hoc analysis with a Tukey’s HSD test. A post-hoc Tukey’s HSD test was only carried if ANOVA provided a K value of more than 2 and analysis of variance gave a significant F-ratio. A p value of less than 0.05 was considered to be statistically significant.

## Reagents

**Fly Strains**

**Table:** 

Name	Genotype	Stock ID
*elav-GAL4*	*P{w[+mW.hs]=GawB}elav[C155]*	BDSC 458
*GMR-GAL4*	w[*]; P{w[+mC]=GAL4-ninaE.GMR}12	BDSC 1104
*hzg::GFP*	y[1] w[*]; Mi{PT-GFSTF.1}hzg[MI02112-GFSTF.1]	BDSC 63163
*UAS-deGradFP*	y[1] w[*]; P{w[+mC]=UAS-Nslmb-vhhGFP4}2	BSDC 38422
*UAS-hzg RNAi1*	*w[1118]; P{GD11782}v40611 CG5830*	VDRC 40611
*UAS-hzg RNAi2*	*w[1118]; P{attP,y[+],w[3`v101539] CG5830*	VDRC 101539
BDSC, Bloomington Drosophila Stock Center; VDRC, Vienna Drosophila Resource Center.		
**Antibodies**		
Name	Concentration	Catalogue Number and Source
Fasciclin II (Fas2)	1:20	1D4, DSHB
Bruchpilot	1:100	nc82, DSHB
Alpha-tubulin	1:500	12G10, DSHB
GFP	1:20,000	Ab260, Abcam
Goat anti-mouse Alexa 555	1:500	A24122, Sigma Aldrich
Goat anti-rabbit Alexa 647	1:500	A21244, Sigma Aldrich
ECL Mouse IgG HRP-linked	1:20,000	NA931VS, GE Healthcare
ECL Rabbit IgG HRP-linked	1:20,000	NA934VS, GE Healthcare
DSHB, Developmental Studies Hybridoma Bank.		
